# Vietnamese version of the Hypertension Knowledge-Level Scale (HK-LS): Translation and validation

**DOI:** 10.34172/jcvtr.31855

**Published:** 2024-03-13

**Authors:** Thao Huong Nguyen, Hanh Hong Nguyen, Anh Mai Huynh, Thanh Van Vo, Han Diep Gia, Thang Nguyen

**Affiliations:** ^1^Department of Clinical Pharmacy, University of Medicine and Pharmacy at Ho Chi Minh City, Ho Chi Minh City 81000, Vietnam; ^2^Faculty of Pharmacy, Can Tho University of Medicine and Pharmacy, Can Tho City 900000, Vietnam; ^3^Department of Pharmacology and Clinical Pharmacy, Can Tho University of Medicine and Pharmacy, Can Tho City 900000, Vietnam

**Keywords:** Hypertension, Questionaire, Translation, Validation, Vietnamese

## Abstract

**Introduction:**

In Vietnam, the prevalence of hypertension is increasing rapidly. Patients need to be conscious of the disease for timely prevention and treatment. The Hypertension Knowledge Level Scale (HK-LS) is commonly used to assess knowledge about hypertension.

**Methods:**

Data collection was took place in a hospital in Binh Thuan province, Vietnam in February 2020 with a total of 184 paticipants. Translation and adaptation of the HK-LS, validate the questionnaire through in-person interviews with outpatients diagnosed with hypertension. The translation process followed WHO guidelines. The appraisal process evaluates through reliability (Cronbach’s alpha coefficient) and validity (meaningful relationship between the response results of the scale and the patient’s characteristics).

**Results:**

The Vietnamese version of the HK-LS was translated and proven to be reliable (Cronbach’s alpha=0.72) and valid (statistically significant difference between age groups (*P*=0.021) and educational background (*P*=0.007).

**Conclusion:**

The HK-LS was translated from English into Vietnamese; the questions are clear, intelligible, and suitable for surveying patients in Vietnam.

## Introduction

 One of the major hazards for fatal cardiovascular events such as stroke, myocardial infarction, heart failure, renal failure, etc., is hypertension^1^. It is predicted that by 2025, the number of hypertensive patients will be 1.56 billion.^1^ A research by Sarki Ahmed indicated that 1 in 3 adults has hypertension in developing countries, and hypertension considerably burdens public health in low- and middle-income nations.^2^ In Vietnam, the rate of hypertension is increasing rapidly; the rate in adults is 25.1%.^3^ In addition, a recent study in 2019 by Lana Meigari et al on the rate of hypertension in Vietnam - a systematic review and meta-analysis showed the prevalence of hypertension to be 21.1% (confidence interval 95% = 18.5 - 23.7).^4^

 In a study by Karaeren, knowledge about the causes of hypertension and reasons for needing medication to lower blood pressure (BP) have a good correlation with medication adherence.^5^ Another study showed that most patients achieved a moderate level of knowledge, but the majority of patients incorrectly answered questions about normal BP values and target BP values; they also thought that only taking medication could control BP. More than 95% did not know the name of their antihypertensive medication, but 99.7% could identify the medication they were taking from the investigator’s sample medication packets.^6^ The HK-LS questionnaire assessed the following factors: definition, treatment, adherence, lifestyle, diet, and complications.^7^ The therapist will be able to gauge the patient’s comprehension by asking them questions regarding the ailment they are suffering with. They implement the appropriate educational interventions to improve patient awareness and accomplish treatment targets.

 The HK-LS has been used in studies performed on different populations to determine knowledge of hypertension and risk factors.^8-10^ Nevertheless, it has not been validated and reliable in Vietnam.^11,12^ We now understand how crucial it is to translate and validate the Vietnamese version of the questionnaire to measure hypertensive patients’ understanding of their disease condition. Thus, we carried out this study to translate, adapt, and validate the HK-LS for use in Vietnam.

## Materials and Methods

###  Study participants 

 The study was conducted on outpatient consulting at Southern Regional General Hospital, Vietnam. A pilot study involving 30 participants was conducted from February 2 to 6, 2020, while questionnaire validation undertaken by 154 participants was conducted from February 7 to March 29, 2020.

 Inclusion criteria: 1) were 18 years old or older; 2) were outpatients with a diagnosis of hypertension; 3) were treated with at least one drug for three months or more; 4) could hear, speak, and answer the question.

 Exclusion criteria: 1) patients refused to participate in the study; 2) the patient was not Vietnamese; 3) the patient was participating in another intervention-related study on knowledge and antihypertensive medication.

###  Methods

 We conducted a study on the HK-LS, the questionnaire consisted of 22 questions, and each question had 3 options (True, False, Unknown). 1 point was scored for each correct answer, and 0 points for the incorrect choice; the patient’s hypertension knowledge was assessed based on the total score.

####  Translation and adaptation of the Hypertension Knowledge Level Scale

 We translated and adapted the HK-LS according to WHO guidelines,^13^ including 5 steps as follows:

1. Initial translation: The HK-LS was translated from English (Supplementary Table S1) into Vietnamese by a Vietnamese person who is proficient in English. The translator had medical expertise and comprehended the study objective and content. We expected the result to be an initial translation, a report detailing the difficulties encountered by the translator.
2. Expert panel: The expert committee included 5 members: 1 translator from the initial translation step, 1 doctor with expertise in the study field, 2 pharmacists with expertise in the study field, and 1 expert in the study method. Experts addressed wordings, incomplete concepts, and translation differences from the existing or original versions. The result obtained would be the pilot version.
3. Back translation: A translator was people who could use English fluently, did not know about the questionnaire, and translated the questionnaire from Vietnamese to English. We expected the result to be a back translation.
4. Pre-testing: The participants were invited to in-person interviews through a prepared scale in Vietnamese (Supplementary Table S2). 30 respondents with hypertension who were willing to take the pre-test from various socioeconomic backgrounds were involved in this study.^14^ The interviewers inquired the patient what they thought the question was asking about; could they repeat the question by their own expression, and whether there were any words or expressions that were difficult for the patient to understand. The participants then gave comments on the wording of each sentence in the pilot version. We expected the result to be a compilation of patient opinions.
5. Adaptating and completing the questionnaire: After collecting the sample, the expert panel adapted the questionnaire based on the patient’s opinion; they adjusted the question’s wording to be reasonable. We expected the results to be a complete Vietnamese version of the questionnaire.

####  Validation of the HK-LS 

 We conducted a cross-sectional study on 154 patients. This was based on the recommendation from Terwee et al, the minimum sample size to validate the scale was 50 patients.^15^ We collected data for a week, 154 patients who met the inclusion criteria were involved in this study. In our study, we collected data through in-person interviews with patients. Patients provided written informed consent prior to participating in the study. Then face-to-face interview with a pretested structured questionnaire was conducted to collect the patients’ characteristics, and hypertension knowledge of patients. The in-person interview was employed using five trained BSc graduate nurses who were working out of the chronic illness clinics.

###  Statistical analysis

 After collecting samples, the data were processed using Excel 2010 and SPSS 25.0 software. We applied descriptive statistics to calculate the frequency and percentage of the characteristics of the study population, knowledge, and medication adherence. Descriptive statistics were also used to calculate the mean and standard deviation to derive the range of patients ages enrolled in this study. Mann-Whitney and Kruskal-Wallis tests were used to determine the differences in the median knowledge scores of age group, gender, occupation, duration of hypertension, and education level. A *P* value < 0.05 was considered statistically significant.

 We tested the reliability of the Vietnamese version through internal consistency. Internal consistency was based on Cronbach’s alpha coefficient value which is higher than 0.7 is consistent.^16^ Construct validity was used to assess the version’s validity, it was investigated based on the significant differences between the results of the scale and the patient’s characteristics.^17^

## Results

###  Initial translation and opinion of the expert panel

 We completed the initial translation (Supplementary Table S3) along with a report of the translator’s difficulties and the expert panel’s opinion (Table 1). In sentence 4, the expert panel recommended translating it as “If the use of the medication for increased blood pressure” instead of the translator’s translation. After discussion, the expert panel decided to use both translations for the pilot study. In sentence 6, translating “feel good” as “feel good” would be verbatim, but it would not be appropriate in the context of this question. Therefore, after discussion, the expert panel agreed to adjust sentence 6 as follows: “in the way they think is best”. For sentence 17, the expert panel recommended translating the phrase “alcoholic beverages” as “alcoholic beverages”. The expert panel agreed to use both translations for the pilot study to select the most optimal translation.

**Table 1:** Opinion of the expert panel for the HK-LS

**Questionnaire item**	**Difficulty**	**Recommendations of the expert panel**
High diastolic or systolic blood pressure indicates increased blood pressure	Expression of the word “indicates”	We translated “indicates” into “inform” in Vietnamese
Increased diastolic blood pressure also indicates increased blood pressure	Expression of the word “indicates”	We translated “indicates” into “inform” in Vietnamese
Increased diastolic blood pressure is the result of aging, so treatment is unnecessary	Expression of the phrase “the result of aging”	We translated “the result of aging” into “due to aging” in Vietnamese
If the medication for increased blood pressure can control blood pressure, there is no needs to change the lifestyle	No problem	4a. “If the medication for increased blood pressure”
		4b. “If the use of the medication for increased blood pressure”
6. Individuals with increased blood pressure must take their medication in a manner that makes them feel good	Expressions of the phrase “in a manner that makes them feel good”	In Vietnamese, we translate that phrase into “in the way they think is best”
Drugs for increased blood pressure must be taken every day	No difficulty	We translated “every day” into “daily” in Vietnamese
For individuals with increased blood pressure, the best cooking method is frying	No difficulty	We translate that phrase into “how to cook food” in Vietnamese
For individuals with increased blood pressure, the best cooking method is boiling or grilling	No difficulty	We translate that phrase into “how to cook food” in Vietnamese
Individuals with increased blood pressure can eat salty foods as long as they take their drugs regularly	No difficulty	we translate “eat salty foods” into “eat salty” in Vietnamese
13. Individuals with increased blood pressure must eat fruit and vegetables frequently	No difficulty	We translated “must” into “need to” in Vietnamese
16. Individuals with increased blood pressure must not smoke	No difficulty	We translate that phrase into “do not need to” in Vietnamese
17. Individuals with increased blood pressure can drink alcoholic beverages	No difficulty	17a. “alcohol/beer”
		17b. “alcoholic beverages”
19. Increased blood pressure can cause heart diseases, such as heart attack if left untreated	No difficulty	We translated “heart” into “on heart” in Vietnamese

HK-LS: Hypertension Knowledge-Level Scale

###  The pilot study results

 There were 30 participants (mean age = 58.73 ± 9.5), and 63.3% of patients had comorbidities. The proportion of patients with hypertension from less than 5 years was (56.7%), and those with primary education or less was 43.3%. The results are presented in Table 2.

**Table 2:** Characteristics of patients participating in the pilot study

**Characteristics**		**Number** **(N=30)**	**%**
Gender	Female	16	53.3
	Male	14	46.7
Age	Mean ± SD	58.73 ± 9.5	
Comorbidities	Yes	19	63.3
	No	11	36.7
Duration of hypertension	< 5 years	17	56.7
	≥ 5 years	13	43.4
Educational level	≤ Primary school	13	43.3
	≥ Secondary school	17	56.7

###  Adaptation the HK-LS after the pilot study

 In sentence 4, 30 patients understood the meaning of the two options; all patients understood “drug” to mean “drug to treat hypertension”. However, 21 patients thought that option 4b was easier to understand. In sentences 14 and 15, many patients misunderstand “red meat” and “white meat.” To ensure that the patients understood the same problem during the pilot, the expert panel recommended adjusting the translation of sentence 14 to “The best type of meat for individuals with increased blood pressure is red meat, for example, pork, beef, lamb…” and sentence 15 to “The best type of meat for individuals with increased blood pressure is white meat, for example, chicken, fish…”. For question 17, all 30 patients understood both sentences 17a and 17b. Therefore, the expert panel chose option 17b to be closer to the original. The patients’ opinions are shown in Table 3.

**Table 3:** Patients' opinions in the pilot study and adjustment of the expert panel

**Sentence**	**Test translation**	**Patient's opinion**	**Completed Vietnamese version**
4	4a. If the medication for increased blood pressure can control blood pressure, lifestyle changes are not necessary	30 patients understood both sentences. However, 21 patients thought that sentence 4b was easier to understand. In the interview context, all patients understood “Drug” as “drug to treat hypertension”.	If the medication can control blood pressure, there is no needs to change lifestyle
	4b. If the use of the medication for increased blood pressure can control blood pressure, no lifestyle changes are needed		
14	The best type of meat for individuals with increased blood pressure is red meat	7 patients chose the answer “Wrong” (the correct answer). However, in those 7 patients, 2 patients considered red meat as raw/rare meat, and 3 considered red meat as lean meat. 8 patients did not know what red meat was, including 1 patient who knew that beef was not good for patients with hypertension.	The best type of meat for individuals with increased blood pressure is red meat, for example, pork, beef, lamb, etc.
15	The best type of meat for individuals with increased blood pressure is white meat	3 patients thought that white meat was fatty meat, and 9 patients thought that pork was white meat.	The best type of meat for individuals with increased blood pressure is white meat, for example, chicken, fish, etc.
17	17a. Individuals with increased blood pressure can drink alcohol/beer	All 30 patients understood both sentences	Individuals with increased blood pressure can drink alcoholic beverages
	17b. Individuals with increased blood pressure can drink alcoholic beverages		

###  Validation of the HK-LS

####  Patients’ characteristics

 This study involved 154 patients (mean age = 60.5 ± 9.0), and 61.7% of patients had education level from secondary above. The proportion of patients with hypertension from less than 5 years was 53.9%, and 48.7% of patients had comorbidities (Table 4).

**Table 4:** Characteristics of patients participating in the validation

**Characteristics**		**Number** **(N=154)**	**%**
Age	Mean ± SD	60.5 ± 9.0	
Age group	< 65 years old	101	65.5
	≥ 65 years old	53	34.5
Gender	Female	72	46.8
	Male	82	53.2
Occupation	Working	82	53.2
	Not working	72	46.8
Educational level	≤ Primary school	59	38.3
	≥ Secondary school	95	61.7
Duration of hypertension	< 5 years	83	53.9
	≥ 5 years	71	46.1
Comorbidities	Yes	75	48.7
	No	79	51.3

####  Reliability

 The Cronbach’s alpha coefficient for the total scale was 0.72, ranging from 0.7 to 0.9, and all the Corrected Item - Total Correlation was greater than 0.3 (Table 5).

**Table 5:** Cronbach's alpha coefficient value of the HK-LS

**Sub-dimension item**	**Item**	**Corrected Item - Total Correlation**	**Cronbach's alpha for each sub-dimension item**	**Cronbach's alpha of the total scale**
Definitions	1	0.84	0.91	0.72
	2	0.84		
Medical treatment	6	0.38	0.66	
	7	0.49		
	8	0.47		
	9	0.50		
Medicine Adherence	3	0.43	0.65	
	4	0.37		
	5	0.45		
	12	0.47		
Life style	10	0.39	0.59	
	11	0.37		
	13	0.32		
	16	0.31		
	17	0.38		
Diets	14	0.56	0.72	
	15	0.56		
Complications	18	0.51	0.72	
	19	0.49		
	20	0.48		
	21	0.41		
	22	0.55		

HK-LS: Hypertension Knowledge-Level Scale

####  Construct validity

 We recorded a statistically significant difference (*P* < 0.05) in the proportion of patients with good knowledge and poor knowledge between age group characteristics (* P* = 0.021) and level of education (*P* = 0.007), proving that the HK-LS scale achieved the construct validity (Table 6).

**Table 6:** The relationship between patient characteristics and knowledge about hypertension

**Characteristics**		**Poor knowledge**		**Good knowledge**		***p***
		**Number** **(N=94)**	**%**	**Number** **(N=60)**	**%**	
Age group	< 65 years old	55	35.71	46	29.87	0.021
	≥ 65 years old	39	25.33	14	9.09	
Gender	Female	43	27.92	29	18.83	0.754
	Male	51	33.12	31	20.13	
Occupation	Working	42	27.27	30	19.48	0.519
	Not working	52	33.77	30	19.48	
Educational level	≤ Primary school	44	28.57	15	9.74	0.007
	≥ Secondary school	50	32.47	45	29.22	
Duration of hypertension	< 5 years	55	35.71	28	18.18	0.150
	≥ 5 years	39	25.32	32	20.78	
Comorbidities	Yes	43	27.92	32	20.78	0.358
	No	51	33.12	28	18.18	

####  The Vietnamese version of the Hypertension Knowledge Level Scale

 Supplementary Table S4 presents the translated and reliable proved HK-LS (Cronbach’s alpha = 0.72) and it had the construct validity (there was a statistically significant difference in knowledge between age groups and education level).

## Discussion

 During the translation and cross-cultural adaptation process, the scale was considered to adjust to fit with the Vietnamese context. At this stage, in sentence 3, to make it easier for patients to understand the phrase “the result of aging”, the expert panel recommended adjusting it to “due to aging”. In keeping with the context, the phrase “feel good” in sentence 6 was translated as “considered the best” in Vietnamese. This translation was also consistent with the Brazilian translation.^18^ The translation was not simply literal but had to be based on each context and sentence to make the appropriate adjustment and make it easy to understand. In sentence 7, the expert panel proposed to change the phrase “every day” with “daily” to be closer and more common to the patient. The expert panel thought that the words “must” in sentence 13 and “must not” in sentence 16 should be translated as “cần phải” and “không được” in the Vietnamese version instead of “nên” and “không nên” like the initial translation. For sentence 17, the expert panel proposed to translate the phrase “alcoholic beverages” as “alcoholic beverages” because in addition to “alcohol/beer” there were many other types of alcoholic beverages. After discussion, the expert panel agreed to use both translation options for the pilot study.

 In the pilot translation with sentences 4 and 17, there were 2 options; all the participating patients understood both translations. In sentence 4, most patients thought option 4b was easier to understand. Using both options for the pilot translation was optimal to make the complete translation more suitable for most patients. We recorded patients who accidentally chose the correct answer despite not having true knowledge. Specifically, in sentences 14, and 15, the expression “red meat” and “white meat” caused misunderstandings for the patient. Significant challenges were the different languages and expressions. Clarifying the concept of “red meat” and “white meat” aimed to help all patients when asked to understand the problem equally, avoiding mistakes when surveying. Consequently, every single stage of this study aimed to customize the tool for the adult Vietnamese population, with the ultimate goal being to create a sufficient version that could be utilized by individuals from a wide range of sociocultural backgrounds.

 Regarding reliability, the Cronbach’s alpha coefficient of each aspect: “definition”, “treatment”, “adherence”, “lifestyle”, “diet”, and “complications” were respectively 0.91, 0.66, 0.65, 0.59, 0.72, 0.72, and the total scale was 0.72. Similar results of Erkoc et al were 0.82 for the total scale, 0.92 for “definition”, 0.59 for “treatment”; 0.67 for “adherence”, 0.77 for “lifestyle”, 0.72 for “diet” and 0.76 for “complications”.^7^ We found that the Cronbach’s alpha values in the aspect of “definition”, “adherence”, and “diet” in our study were quite similar to the results of Erkoc’s study. Although our Vietnamese version’s Cronbach’s alpha coefficient was lower than that of Erkoc’s study, it was still within the range of 0.7 to 0.9. This showed that the scale had a good consistency. In a similar study of translation and validation of the Greek version of the HK-LS scale, the Cronbach`s alpha coefficient was 0.66.^9^ This value in the Brazilian study on the association between patient knowledge and medication adherence among hypertensive patients was 0.92.^18^ This difference can be explained by two following reasons: 1) the Vietnamese version of the HK-LS scale was adapted to suit Vietnam’s cultural - economic - social conditions. For example, questions 14, and 15 had been modified to be more patient-appropriate. 2) Cronbach’s alpha coefficient was representative of the pilot population.^20,21^ For example, in the meta-analytical study of Caruso (2000) on satisfaction measurement, the Cronbach’s alpha coefficient was 0.79 in the general population, but in the clinician population, this value only reached 0.62.^22^ All validation studies showed that the HK-LS scale had a good consistency. Besides determining Cronbach’s alpha coefficient, we also determined the Corrected Item - Total Correlation. The coefficient of all the questions was higher than 0.3. This showed that all questions contributed to measuring the patient’s knowledge.^21^

 Regarding the construct validity, the results demonstrated a statistically significant difference in knowledge between age groups (*P* = 0.021) and education level groups (*P* = 0.007), showing the structural value of the questionnaire. Patients with greater education levels—high school or above—had better knowledge about hypertension than patients with lower education. In addition, patients under 65 years old had better hypertension knowledge than the elderly (*P* = 0.021). These findings revealed that to increase patient comprehension and improve treatment efficacy, more attention should be allocated to informing patients about diseases, particularly those with lower educational backgrounds and elderly patients.

 Our study has several limitations. Firstly, the way to evaluate the score is not clear and objective; in the scale, there were 22 questions, and for each question, there would be 3 options (true, false, unknown). Nevertheless, there were only points for correct answers and no points for incorrect answers, so the unknown choice did not clarify how the points were calculated. We suggested that a score of 0 could be calculated for the unknown choice. Secondly, in the knowledge scale about hypertension, knowledge about the causes of hypertension was also crucial; this was a shortcoming that needed to be supplemented.

 The scale met the reliability and construct validity criteria and could be used as a measurement tool in medication adherence studies. We hoped our validation translation was the basis for future studies. It could be applied to find the relationship between knowledge and adherence to blood pressure goals. Further studies should be conducted on interventions focusing on improving knowledge about hypertension, thereby providing the right knowledge for patients and achieving good results in treatment.

## Conclusion

 The Hypertension Knowledge Scale was translated from English into Vietnamese. The Vietnamese version of the scale consists of 22 questions. All questions were clear, easy to understand, and suitable for surveying patients in Vietnam. The reliability of the HK-LS scale in the Vietnamese version had a good consistency with Cronbach’s alpha coefficient of the total scale of 0.72 and reached construct validity. We proposed to conduct an intervention study to improve the knowledge of hypertensive patients, help provide the right knowledge to the patient, and achieve a good treatment effect.

## Acknowledgments

 We would like to thank all colleagues and medical staff at the hospital for supporting us in this research.

## Competing Interests

 None to declare.

## Ethical Approval

 Our research strictly adheres to ethical criteria in medical research and was approved by the Southern Regional General Hospital Council with decision No.20200115. Information about participating patients was kept confidential. This study did not affect the customs and traditions of the research subjects. We always respected the patient’s voluntary right; during the study, the patient had the right to refuse to participate at any time.

## Funding

 The authors received no financial support for the research, authorship, and/or publication of this article.

## Supplementary Files

Supplementary File contains Table S1-S4.
