# Supplementary material for: Vietnamese version of the Hypertension Knowledge-Level Scale (HK-LS): Translation and validation
Source: J Cardiovasc Thorac Res. 2024 Mar 13;16(1):38–44. doi: 10.34172/jcvtr.31855 (PMC10997980; doi:10.34172/jcvtr.31855)
Supplement: Supplementary file 1 — Supplementary File contains Table S1-S4. [file jcvtr-16-38-s001.pdf]

J Cardiovasc Thorac Res, 2024, 16(1), 38-S1
doi: 10.34172/jcvtr.2024.31855
http://jcvtr.tbzmed.ac.ir

Table S1. The Hypertension Knowledge Level Scale (HK-LS) - original version

Item	Content
1	High diastolic or systolic blood pressure indicates increased blood pressure
2	Increased diastolic blood pressure also indicates increased blood pressure
3	Increased diastolic blood pressure is the result of aging, so treatment is unnecessary
4	If the medication for increased blood pressure can control blood pressure, there is no need to change lifestyle
5	If individuals with increased blood pressure change their lifestyle, there is no need to treatment
6	Individuals with increased blood pressure must take their medication in a manner that makes them feel good
7	Drugs for increased blood pressure must be taken every day
8	Individuals with increased blood pressure must take their medication only when they feel ill
9	Individuals with increased blood pressure must take their medication throughout their life
10	For individuals with increased blood pressure, the best cooking method is frying
11	For individuals with increased blood pressure, the best cooking method is boiling or grilling

12	Individuals with increased blood pressure can eat salty foods as long as they take their drugs regularly
13	Individuals with increased blood pressure must eat fruit and vegetables frequently
14	The best type of meat for individuals with increased blood pressure is red meat
15	The best type of meat for individuals with increased blood pressure is white meat
16	Individuals with increased blood pressure must not smoke
17	Individuals with increased blood pressure can drink alcoholic beverages
18	Increased blood pressure can cause strokes, if left untreated
19	Increased blood pressure can cause heart diseases, such as heart attack, if left raw
20	Increased blood pressure can cause premature death if left untreated
21	Increased blood pressure can cause kidney failure, if left untreated
22	Increased blood pressure can cause visual disturbances, if left untreated

Table S2. Questionnaire on hypertension knowledge (the pilot version)

Dear Sir/Madam, we are a study team from the Southern Regional General Hospital of Binh Thuan Province, conducting a patient information survey to study knowledge and adherence to medication in hypertensive patients. Outpatient pressure at the Southern Regional General Hospital of Binh Thuan province in 2020.

Please answer the questions listed below. The information you provide will be used for study purposes.

Thank you for agreeing and taking the time to participate in the survey. We assure you that the personal information you provide will be kept confidential.

PART 1. BASIC INFORMATION

1.1.	Patient name:	
1.2.	Year of Birth	
1.3.	Gender	□ Male □ Female
1.4.	Education level	□ Below primary school<br>□ Primary School<br>□ Secondary School<br>□ High School<br>□<br>Postgraduate/University/College/Intermediate
1.5.	Where do you live?	Binh Thuan<br>□ Neighboring provinces of Binh Thuan<br>□ Other provinces: ……………………
1.6.	Marital status	□ Single<br>□ Married
1.7.	Employment	□ Working<br>□ Not working
1.8.	Duration of hypertension	□ < 5 years □ ≥ 5 years
1.9	Chronic comorbidities	□ Yes □ No

PART 2. KNOWLEDGE QUESTIONS

**Sentence**	**Content**	**True**	**False**	**Unknown**

**1**	Huyết áp tâm trương hoặc huyết áp tâm thu cao cho biết bị tăng huyết áp			
**2**	Huyết áp tâm trương cao cũng cho biết bị tăng huyết áp			
**3**	Huyết áp tâm trương tăng là do sự lão hóa, vì vậy không cần phải điều trị			
**4a**	Nếu thuốc điều trị tăng huyết áp có thể kiểm soát huyết áp, thì không cần phải thay đổi lối sống			
**4b**	Nếu việc dùng thuốc điều trị tăng huyết áp có thể kiểm soát huyết áp thì không cần phải thay đổi lối sống.			
**5**	Nếu những người bị tăng huyết áp thay đổi lối sống, thì không cần phải điều trị			
**6**	Những người bị tăng huyết áp cần phải dùng thuốc theo cách mà họ cho là tốt nhất			
**7**	Thuốc điều trị tăng huyết áp phải được uống hàng ngày			
**8**	Những người bị tăng huyết áp chỉ phải uống thuốc khi cảm thấy bị bệnh			
**9**	Những người bị tăng huyết áp cần phải dùng thuốc suốt đời			
**10**	Cách chế biến thức ăn tốt nhất cho những người bị tăng huyết áp là chiên/ xào.			

**11**	Cách chế biến thức ăn tốt nhất cho những người bị tăng huyết áp là luộc hoặc nướng			
**12**	Những người bị tăng huyết áp có thể ăn mặn miễn là họ uống thuốc thường xuyên			
**13**	Những người bị tăng huyết áp cần phải ăn trái cây và rau củ thường xuyên			
**14**	Loại thịt tốt nhất cho những người bị tăng huyết áp là thịt đỏ			
**15**	Loại thịt tốt nhất cho những người bị tăng huyết áp là thịt trắng			
**16**	Những người bị tăng huyết áp không được hút thuốc			
**17a**	Những người bị tăng huyết áp có thể uống rượu/bia			
**17b**	Những người bị tăng huyết áp có thể uống thức uống có cồn			
**18**	Nếu không được điều trị, tăng huyết áp có thể gây đột quỵ			
**19**	Nếu không được điều trị, tăng huyết áp có thể gây ra các bệnh trên tim, như nhồi máu cơ tim			
**20**	Nếu không được điều trị, tăng huyết áp có thể gây tử vong sớm			

**21**	Nếu không được điều trị, tăng huyết áp có thể gây suy thận			
**22**	Nếu không được điều trị, tăng huyết áp có thể gây rối loạn thị giác			

Table S3. The initial translation

Item	Content
1	Huyết áp tâm trương hoặc huyết áp tâm thu cao cho thấy bị tăng huyết áp
2	Huyết áp tâm trương cao cũng cho thấy bị tăng huyết áp
3	Huyết áp tâm trương tăng là kết quả của tuổi già, vì vậy điều trị là không cần thiết
4	Nếu thuốc điều trị tăng huyết áp có thể kiểm soát huyết áp, thì không cần phải thay đổi lối sống
5	Nếu những người bị tăng huyết áp thay đổi lối sống, thì không cần phải điều trị
6	Những người bị tăng huyết áp cần phải dùng thuốc theo cách mà họ cảm thấy dễ chịu
7	Thuốc điều trị tăng huyết áp phải được uống mỗi ngày
8	Những người bị tăng huyết áp chỉ phải dùng thuốc khi cảm thấy bị bệnh
9	Những người bị tăng huyết áp phải dùng thuốc suốt đời
10	Cách nấu ăn tốt nhất cho những người bị tăng huyết áp là chiên.
11	Cách nấu ăn tốt nhất cho những người bị tăng huyết áp là luộc hoặc nướng

10	Những người bị tăng huyết áp có thể ăn thức ăn mặn miễn là họ uống thuốc thường xuyên
13	Những người bị tăng huyết áp nên ăn trái cây và rau củ thường xuyên
14	Loại thịt tốt nhất cho những người bị tăng huyết áp là thịt đỏ
15	Loại thịt tốt nhất cho những người bị tăng huyết áp là thịt trắng
16	Những người bị tăng huyết áp không nên hút thuốc
17	Những người bị tăng huyết áp có thể uống rượu/bia
18	Tăng huyết áp có thể gây đột quỵ nếu không được điều trị
19	Tăng huyết áp có thể gây ra các bệnh về tim, chẳng hạn như nhồi máu cơ tim, nếu không được điều trị
20	Tăng huyết áp có thể gây tử vong sớm nếu không được điều trị
21	Tăng huyết áp có thể gây suy thận, nếu không được điều trị
22	Tăng huyết áp có thể gây rối loạn thị giác, nếu không được điều trị

Supplementary S4. The Vietnamese version of the HK-LS

Item	Vietnamese version
1	Huyết áp tâm trương hoặc huyết áp tâm thu cao cho biết bị tăng huyết áp.
2	Huyết áp tâm trương cao cũng cho biết bị tăng huyết áp.
3	Huyết áp tâm trương tăng là do sự lão hóa, vì vậy không cần phải điều trị.
4	Nếu việc dùng thuốc có thể kiểm soát huyết áp thì không cần phải thay đổi lối sống.
5	Nếu những người bị tăng huyết áp thay đổi lối sống, thì không cần phải điều trị.
6	Những người bị tăng huyết áp cần phải dùng thuốc theo cách mà họ cho là tốt nhất.
7	Thuốc điều trị tăng huyết áp phải được uống hàng ngày.
8	Những người bị tăng huyết áp chỉ phải dùng thuốc khi cảm thấy bị bệnh.
9	Những người bị tăng huyết áp cần phải dùng thuốc suốt đời.
10	Cách chế biến thức ăn tốt nhất cho những người bị tăng huyết áp là chiên/ xào.
11	Cách chế biến thức ăn tốt nhất cho những người bị tăng huyết áp là luộc hoặc nướng.
12	Những người bị tăng huyết áp có thể ăn mặn miễn là họ uống thuốc thường xuyên.
13	Những người bị tăng huyết áp cần phải ăn trái cây và rau củ thường xuyên.
14	Loại thịt tốt nhất cho những người bị tăng huyết áp là thịt đỏ, ví dụ: thịt heo, thịt bò, thịt cừu…
15	Loại thịt tốt nhất cho những người bị tăng huyết áp là thịt trắng, ví dụ: thịt gà, cá…
16	Những người bị tăng huyết áp không được hút thuốc.
17	Những người bị tăng huyết áp có thể uống thức uống có cồn.
18	Nếu không được điều trị, tăng huyết áp có thể gây đột quỵ.

19	Nếu không được điều trị, tăng huyết áp có thể gây ra các bệnh trên tim, như nhồi máu cơ tim.
20	Nếu không được điều trị, tăng huyết áp có thể gây tử vong sớm.
21	Nếu không được điều trị, tăng huyết áp có thể gây suy thận.
22	Nếu không được điều trị, tăng huyết áp có thể gây rối loạn thị giác.
Evaluation method: each correct answer will get 1 point, and each incorrect answer will get 0 points.<br>Correct: 1, 2, 7, 9, 11, 13, 15, 16, 18 – 22;<br>Incorrect: 3 – 6, 8, 10, 12, 14, 17.<br>Total accumulated points from 0 to 22 points.<br>Poor knowledge: 0-17 points.<br>Good knowledge: 18-22 points.	

HK-LS: Hypertension Knowledge-Level Scale
